# Integrated Diagnostic Framework for Process and Sensor Faults in Chemical Industry

**DOI:** 10.3390/s21030822

**Published:** 2021-01-26

**Authors:** Jiaxin Zhang, Wenjia Luo, Yiyang Dai

**Affiliations:** 1 School of Chemical Engineering, Sichuan University, Chengdu 610065, China; 201821000250@stu.swpu.edu.cn; 2 School of Chemistry and Chemical Engineering, Southwest Petroleum University, Chengdu 610500, China; luowenjia@swpu.edu.cn

**Keywords:** process and sensor fault, cycle temporal algorithm, dynamic kernel principal component analysis, reconstruction-based contribution, integrated diagnostic framework

## Abstract

This study considers the problem of distinguishing between process and sensor faults in nonlinear chemical processes. An integrated fault diagnosis framework is proposed to distinguish chemical process sensor faults from process faults. The key idea of the framework is to embed the cycle temporal algorithm into the dynamic kernel principal component analysis to improve the fault detection speed and accuracy. It is combined with the fault diagnosis method based on the reconstruction-based contribution graph to diagnose the fault variables and then distinguish the two fault types according to their characteristics. Finally, the integrated fault diagnosis framework is applied to the Tennessee Eastman process and acid gas absorption process, and its effectiveness is proved.

## 1. Introduction

The main goal of the chemical industry is to improve the efficiency and accuracy of manufacturing facilities. As the automation level of the chemical industry continues to improve, the scale of production increases and the complexity of the system grows continually. The probability of failure and fault in chemical system also increases. Compared with the permanent termination of the system caused by failure, the system offset caused by fault can be detected in time, and then corresponding measures can be taken to avoid accidents [1]. Therefore, distinguishing the type of faults in the chemical process is the key to reducing operator errors and ensuring system safety and reliability.

In recent years, many fault diagnosis methods for chemical processes have emerged. These methods diagnose different faults [2,3,4,5]. However, no distinction is made between the types of faults themselves. In an actual chemical process, faults can be divided into process faults and sensor faults [6]. Process faults are characterised by multivariate coordination. The occurrence of process fault means that the operating state of the system deviates from the normal value. Sensor fault has variable independence, and the fault variable is unique. The sensor fault interferes with the stability of the system and affects the judgment of the operator, which may lead to fault. Therefore, it is very important to diagnose process faults and sensor faults in modern industrial processes [7,8,9].

The fault diagnosis method is the key to ensure safe and effective operation of the process [10]. Generally, fault diagnosis methods can be divided into three categories: model-based [11], knowledge-based [12], and data-based [13] methods. Among them, the principal component analysis (PCA) in data-based methods is widely used for process and sensor fault diagnosis in chemical processes [14,15]. Ku et al., Lee et al., and Yang et al. successively proposed the dynamic principal component analysis (DPCA) [16], kernel principal component analysis (KPCA) [17], and dynamic kernel principal component analysis (DKPCA) [18] for process fault diagnosis. Qin et al. [19] improved the DPCA method and proposed a comprehensive model for sensor fault diagnosis. Wang et al. [20] put forward a new strategy based on PCA for sensor fault diagnosis. Li et al. [21] proposed a sensor fault diagnosis method based on density-based clustering and PCA. When using various PCA models for fault diagnosis, the diagnosis space is divided into two subspaces called principal subspace and residual subspace. $T^{2}$ statistics and squared prediction error (SPE) statistics are constructed to characterise the mean and variance information, respectively, of the processes in the two subspaces [22]. In the process of fault detection using $T^{2}$ and SPE statistics, the following four types of monitoring results may appear: (1) neither of the statistics exceeds the control limit; (2) $T^{2}$ statistics do not exceed the limit, but SPE statistics exceed the limit; (3) $T^{2}$ statistics exceed the limit; and SPE statistics exceed the limit. (4) $T^{2}$ statistics and SPE statistics both exceed the limit. When the SPE statistics change significantly (results (2) and (4)), it means that the normal operating model represented by the PCA model is destroyed, and a process fault or sensor fault has occurred. When the $T^{2}$ statistic changes significantly and the SPE statistic does not change (result (3)), it indicates that the relationship between the variables is approximately satisfied, but the system has undergone some transformation, which may be due to a change in operating conditions or a system fault. Result (1) indicates that the system is normal [23]. According to results (2) and (4), this study makes a detailed distinction between process faults and sensor faults in chemical processes. 

Chemical processes are characterised by large amounts of data and complex calculations. The abovementioned improved methods also have this limitation. To detect the occurrence of faults more rapidly and accurately, the PCA method has been improved accordingly. In temporal logic algorithms [24], the temporal logic can qualitatively describe the behaviour of the system over time, because it has a large number of operators such as ‘always’ and ‘final’. Clarke et al. [25] proposed a computational temporal logic to describe the behaviour of concurrent systems. For chemical processes, a cycle temporal algorithm (CTA) was proposed based on traditional temporal logic to solve a large amount of chemical data and the problem of low calculation accuracy. By combining it with the DKPCA method, it can capture most of the fault information in the system and improve the detection accuracy. the operator can respond to the fault timely and effectively. The fault type is further distinguished through the fault identification step.

When the system detects that a fault has occurred, it needs to locate the current fault location and fault variables, and then determine whether the fault is a process fault or a sensor fault. Currently, the most widely used method for fault variable location is the contribution graph [26]. The method is based on quantifying the contribution of each process variable to a single principal component score. The contribution of each process variable to the main component in the out-of-control state is added, and it is called variable contribution [27,28]. In recent years, many variable identification methods have been reported [29,30,31,32]. This study uses the reconstruction-based contribution (RBC) graph method to reconstruct the faulty variables, emphasises the cross-correlation between variables, and clearly shows the relationship between them. The variable characteristics of different faults are used to determine whether the fault type is a process fault or a sensor fault, so as to provide technical support for the next operation of the subsequent operator.

The current chemical process fault diagnosis methods for process and sensor faults are independent of each other, Sensor faults on non-control loops will not directly cause accidents, but will affect operator judgment and the accuracy of process fault diagnosis. Therefore, it is necessary to distinguish between sensor faults and process faults. A process fault is when the process itself deviates from the normal state, and the sensor fault process itself does not deviate from the normal state, but the sensor index deviates from the true value. Sensor faults on non-control loops will not directly cause accidents, which will affect operator judgment and the accuracy of process fault diagnosis. There is no corresponding integrated model to distinguish process and sensor faults in detail. When a sensor fault occurs in a chemical process, it is regarded as a process fault by default, which could cause operator errors and accidents. Therefore, it is particularly important to distinguish between process faults caused by real fault fluctuations and sensor faults indicated by false data. This paper proposes a CTA to improve the calculation speed and eliminate the redundancy problem caused by big data calculation. The CTA combined with DKPCA improves the fault detection speed and accuracy. Combined with the fault identification model based on the RBC graph, it emphasises the strong correlation of variables while extracting fault information. Finally, an integrated diagnostic framework for distinguishing sensor and process faults in a chemical process is obtained. The framework has the advantages of fast detection speed, high detection accuracy, and accurate fault variables. Its application to the Tennessee Eastman (TE) process and acid gas absorption process proves the effectiveness of the proposed integrated fault diagnosis framework.

The remainder of this paper is structured as follows. In Section 2, the CTA, DKPCA method, and RBC graph are introduced. In Section 3, the method proposed in the previous section is used to build an integrated fault diagnosis framework to distinguish between process and sensor faults. In Section 4, The TE process is compared with other fault detection methods to prove the advantages of the proposed method, and then four faults in the TE process and three faults in the acid gas absorption process are selected to demonstrate the effectiveness of the integrated fault diagnosis framework in distinguishing process and sensor faults. In Section 5, we summarise our work.

## 2. Fault Diagnosis Methods

### 2.1. Dynamic Kernel PCA

Generally, the PCA can only be effectively performed in the observation set of linear and steady-state changes. When the data change nonlinearly and dynamically, the PCA method can be converted to changes in linear data after mapping. However, the analysis method based on the kernel function does not need to calculate the eigenvector as the PCA method but to convert it into the eigenvalue and eigenvector of the kernel matrix. Thus, it avoids the calculation for obtaining the eigenvector in the high-dimensional space and converting it into projection, solving the linear combination of kernel functions, and by capturing the data dynamic matrix [23,24,25,26,27,28,29,30,31,32,33,34,35], it also solves the dynamic matching problem of the PCA model. Hence, the calculation is greatly simplified.

Assuming that the normal data set X contains m variables, and each variable has n observations, the vectors at time t and augmented matrix X(s) containing the observations at the previous s time to reflect the relationship between the variables’ dynamic relationship.

(1) $$X\left(s\right)=\left[\begin{array}{ccc} x_{t}^{T} & \cdots & x_{t-s}^{T}\\ \vdots & \ddots & \vdots \\ x_{t+s-n}^{T} & \cdots & x_{t-n}^{T} \end{array}\right]$$

Then, the dynamic matrix X(s) is used to establish the dynamic principal component model through the PCA method, and then the dynamic characteristics can be analysed.

(2) $$X\left(s\right)=TP^{T}+E$$

where T is the score matrix, P is the load matrix, and E is the residual matrix, which is the projection of the sample in the residual space.

For nonlinear problems, the principle of the KPCA is to map the input data to the high-dimensional feature space through the inner product kernel function Φ to perform the PCA, thereby turning the two linearly inseparable points in the low-dimensional space into linearly separable ones. After nonlinear mapping, the observation vector $x_{i}\left(t:t-s\right)$ is mapped to $\Phi_{i}\left(t:t-s\right)$, and the dynamic data augmentation matrix X(s) is mapped to Φ(s). 

Then, the covariance matrix of the feature space can be expressed as

(3) $$C^{F}=\frac{1}{N}\overset{N}{\underset{i=1}{\sum }}\phi_{i}\left(t:t-s\right)\phi_{i}{}^{T}\left(t:t-s\right)$$

Suppose the eigenvalue λ corresponding to the matrix $C^{F}$, the eigenvector ν, and the coefficient $\alpha_{i}\left(i=1,2,\ldots ,N\right)$ such that.

(4) $$\nu =\overset{N}{\underset{i=1}{\sum }}\alpha_{i}\phi_{i}\left(t:t-s\right)$$

The corresponding characteristic equation is

$$\lambda \nu =C^{F}\nu =\left(\frac{1}{N}\overset{N}{\underset{i=1}{\sum }}\phi_{i}\left(t:t-s\right)\phi_{i}{}^{T}\left(t:t-s\right)\right)\nu$$

(5) $$=\frac{1}{N}\overset{N}{\underset{i=1}{\sum }}\langle \phi_{i}\left(t:t-s\right),\nu \rangle \phi_{i}\left(t:t-s\right)$$

(6) $$\lambda \langle \phi_{k}\left(t:t-s\right),\nu \rangle =\langle \phi_{k}\left(t:t-s\right)\cdot C^{F}\nu \rangle$$

Equations (4) and (6) are combined to obtain:

(7) $$\lambda \overset{N}{\underset{i=1}{\sum }}\alpha_{i}{}^{k}\langle \phi_{k}\left(t:t-s\right),\phi_{i}\left(t:t-s\right)\rangle =\frac{1}{N}\alpha_{i}{}^{k}\langle \phi_{k}\left(t:t-s\right)\cdot \overset{N}{\underset{j=1}{\sum }}\phi_{j}\left(t:t-s\right)\rangle \langle \phi_{j}\left(t:t-s\right),\phi_{i}(t:t-s)\rangle$$

By defining the kernel matrix $K\in R^{N\times N}$**,** $\left[K_{ij}\right]=K_{ij}=\langle \phi_{j}\left(t:t-s\right),\phi_{i}(t:t-s)\rangle$, from the above formula, the feature vector can be obtained as

(8) $$\lambda N\alpha =K_{ij}\alpha$$

where $\alpha ={\left[\alpha_{1},\alpha_{2},\ldots ,\alpha_{N}\right]}^{T}$.

Before adopting the PCA on the feature space F, we first standardise the data, that is, replace K with the following equation:

(9) $$K=K-I_{N}K-KI_{N}+I_{N}KI_{N}$$

where $I_{N}$ is equal to $\frac{1}{N}$ multiplied by an N×N identity matrix $E\in R^{N\times N}$. Therefore, the PCA in the feature space F is equivalent to solving the eigenvalue of Equation (6).

Combining Equations (8) and (4), the eigenvector α of the kernel matrix K can be derived from the eigenvector υ of the matrix $C^{F}$, and it satisfies

(10) $$\langle \nu_{k},\nu_{k}\rangle =1$$

where k=1,2,…,p. p is the number of principals.

By calculating the projection of the mapping data on the feature vector $\nu_{k}$, we find the principal component: $t_{k}=\langle \nu_{k},\phi \left(t:t-s\right)\rangle =\sum_{i=1}^{N}\alpha_{i}^{K}\langle \phi_{i}\left(t:t-s\right),\phi (t:t-s)\rangle =\;\sum_{i=1}^{N}\alpha_{i}^{K}K_{ij}$. 

To solve the eigenvalues of Equation (8), we use $t_{k}$ to calculate the feature space principal vector in the input space and introduce the kernel function $K=\exp\left(-\|x-y\|{}^{2}/2\sigma^{2}\right)$ into the feature space to avoid directly calculating the nonlinear mapping.

### 2.2. Cycle Temporal Algorithm (CTA)

In this section, a new method, the CTA, is proposed, which extends the qualitative trend analysis (QTA) method [36] to temporal constraints, extracts based on temporal series, and calculates the difference between the temporal threshold and the linear fitting error relationship. The linear correlation between variables is preserved, and the principal component is obtained by the subsequent DKPCA method. The correlation saves most of the process differences. Each principal component is a linear combination of all the variables. The data are cyclically segmented and merged, which reduces the amount of calculation, increases the calculation speed, and eliminates the redundancy generated by the calculation of the large data matrix.

The temporal logic equation is summarised and defined as follows:

(11) $$\varphi ≔\left|Τ\right|{\bar{G}}_{\left[b,e\right]}\varphi \left|{\bar{F}}_{\left[b,e\right]}\varphi \right|⊣\varphi \left|\varphi_{1}\wedge \varphi_{2}\right|\varphi_{1}{\bar{U}}_{\left[b,e\right]}\varphi_{2},$$

where Τ is ‘always true’, ‘negation’ (⊣), and ‘combination’ (∧), which are standard Boolean join operators. The time series operators ${\bar{G}}_{\left[b,e\right]}$, ${\bar{F}}_{\left[b,e\right]}$, and ${\bar{U}}_{\left[b,e\right]}$ represent the derived ‘always’, the derived ‘final’, and the derived ‘until’, and [b,e] represents the time interval satisfying b≤e. This study defines u(x[τ]):=f(x[τ])≤0 and $f\left(x\left[\tau \right]\right)={\left(x\left[\tau \right]-k\tau -c\right)}^{2}-\delta_{e}$. $\delta_{e}$ is the preset threshold. Where $\delta_{e}=4\sigma^{2}$, σ is the standard deviation of the data sequence. x[τ] represents the data point at time τ. k and c are constant parameters.

**Figure 1:**
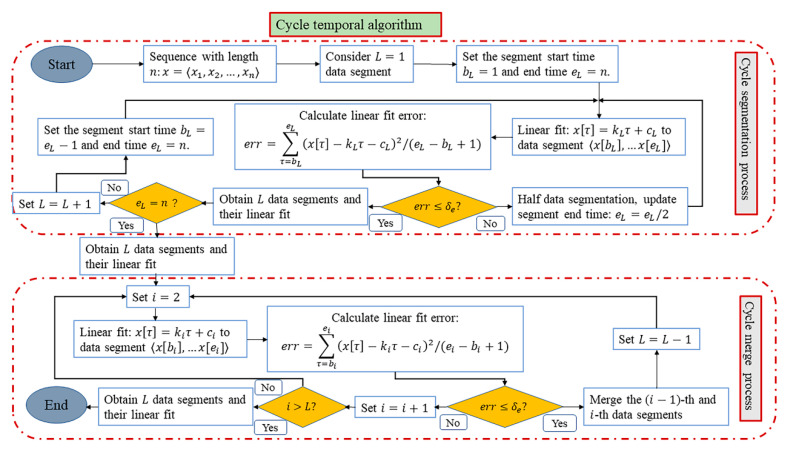
Cycle temporal algorithm (CTA) process.

To eliminate the problem of slow calculation and calculation redundancy in the state of big data, the parameters and thresholds are extracted on the basis of temporal logic, and the data are processed through cycle segmentation and cycle merge. The specific process of the CTA is shown in Figure 1.

The CTA process is divided into a cycle segmentation process and a cycle merge process. For the cycle segmentation process, according to the entire data sequence $x=\langle x_{1},x_{2},\ldots x_{n}\rangle$, L=1 is set as the data segment start, the segment start point $b_{L}$ and the end point $e_{L}$ are set, and a linear fitting is performed on the segment. The fitting equation is $\left(\hat{x}\left[\tau \right]=k_{L}\tau +c_{L}\right)$, where $k_{L}$ and $c_{L}$ represent the slope and the y-axis intercept, respectively. If the linear fitting error err is greater than the extracted threshold $\delta_{e}$, the sequence x is halved. The linear fitting of the first half of the sequence is repeated until the linear fitting error is not greater than the predetermined error threshold $\delta_{e}$. First, it is determined whether the end of the segment reaches the end point, and then, it is determined whether the data are divided into L segments. The linear fitting calculation is expressed in Equation (12).

(12) $$err=\overset{e_{L}}{\underset{L=b_{L}}{\sum }}{\left(x\left[\tau \right]-k_{L}-c_{L}\right)}^{2}/\left(e_{L}-b_{L}+1\right)$$

For the cycle merge process, the obtained L segment data sequence is used as the input into the cycle merge process. First, the data segment is counted by i=2, and the multi-segment data are linearly fitted. Contrary to the cycle segmentation part, when the linear fitting error err is less than the threshold $\delta_{e}$, merge the i−th sequence with the i−1th sequence, then subtract 1 in segments, and merge in turn until the linear fitting error is greater than the threshold $\delta_{e}$. It is judged whether i is greater than L, and if it is, the merge ends.

Based on the above segmented fitting method, cycle segmentation and cycle merge are performed for a given data sequence. A sequence of quaternions of degree L can be obtained.

(13) $$T≔\langle \left(k_{1},c_{1},b_{1},e_{1}\right),\left(k_{2},c_{2},b_{2},e_{2}\right),\ldots ,\left(k_{L},c_{L},b_{L},e_{L}\right)\rangle$$

In each quadruple $\left(k_{i},c_{i},b_{i},e_{i}\right)\left(i\in \left[1,L\right]\right)$, $k_{i}$ and $c_{i}$ are the slope and y-axis intercept of the i-th segment of linear fitting, respectively. The integers $b_{i}$ and $e_{i}$ represent the start and end times of the data segment, respectively, and the corresponding atomic predicate is expressed in Equation (14).

(14) $$u_{i}≔\left({\left(x\left[\tau \right]-k_{i}\tau -c_{i}\right)}^{2}-\delta_{e}\leq 0\right)$$

Based on the above, the temporal logic equation is reconstructed as:

(15) $$\varphi ≔\varphi_{1}U_{\left[b_{1},e_{1}\right]}\varphi_{2}U_{\left[b_{2},e_{2}\right]}\ldots \varphi_{L-1}U_{\left[b_{L-1},e_{L-1}\right]}\varphi_{L}$$

Among them, if i∈[1,L−1], then $\varphi_{i}≔u_{i}$.

Eventually, the CTA is formed. The CTA combines the temporal logic to define the principle of the segmented cycle and determines the size of the cycle, the error threshold, and the final segment starting point and end point. The cycle segmentation of the model data is completed to reduce the dimensionality of the matrix, improving the calculation accuracy and eliminating the calculation redundancy.

### 2.3. RBC Graph

The numerical method of the RBC graph is used to identify the variable contribution rate.

Fault variable reconstruction is used to realise the accurate identification and separation of process and sensor faults by eliminating the influence of observed variables in the fault subspace.

When the system detects a fault $f_{i}$, the observation vector is $x={\left[x_{1},x_{2},\ldots ,x_{m}\right]}^{T}$, where i=1,2,…,m, and m represent the number of process variable observations. The sampling time is omitted here, but the nature of the problem is not changed. If the process vector is reconstructed along the $\zeta_{i}^{T}$ direction, the corresponding relationship is

(16) $$z_{i}=x-\zeta_{i}^{T}f_{i}$$

where $z_{i}$ represents the original value of the process view vector that is not affected by the fault, and Φ is defined as a positive definite symmetric matrix. Then, the joint index $index^{2}\left(z_{i}\right)$ for process monitoring can be obtained:

(17) $$index^{2}\left(z_{i}\right)=z_{i}^{T}\Phi z_{i}=\|z_{i}\|{}_{\Phi }^{2}=\|x-\zeta_{i}^{T}f_{i}\|{}_{\Phi }^{2}$$

When the reconstruction vector $z_{i}$ is infinitely close to or equal to the original value of the observation vector during the normal operation of the process, the joint index in the above equation reaches the minimum, indicating that this is the optimal reconstruction. Therefore, according to Equation (17), the first derivative of the fault parameter is calculated and set to be equal to 0, so that the optimal fault parameter is

(18) $$f_{i}={\left(\zeta_{i}\Phi \zeta_{i}^{T}\right)}^{-1}\zeta_{i}\Phi x$$

From Equations (17) and (18), it can be deduced that the reconstruction contribution of the variable $x_{i}$ to the joint index is

(19) $$RBC_{i}^{index}=\|\zeta_{i}^{T}f_{i}\|{}_{\Phi }^{2}=\|\zeta_{i}^{T}{\left(\zeta_{i}\Phi \zeta_{i}^{T}\right)}^{-1}\zeta_{i}\Phi x\|{}_{\Phi }^{2}=x^{T}\Phi \zeta_{i}^{T}{\left(\zeta_{i}\Phi \zeta_{i}^{T}\right)}^{-1}\zeta_{i}\Phi x$$

Substituting the relationship $\Phi =L\Lambda L^{T}$ into the above equation to restore, the reconstruction contribution of the variable $x_{i}$ to the joint index is obtained as:

(20) $$RBC_{i}=x^{T}L\Lambda L^{T}\zeta_{i}^{T}{\left(\zeta_{i}L\Lambda L^{T}\zeta_{i}^{T}\right)}^{-1}\zeta_{i}L\Lambda L^{T}x=\frac{{\left(\zeta_{i}L\Lambda L^{T}x\right)}^{2}}{{\left(L\Lambda L^{T}\right)}_{i}}$$

where ${\left(L\Lambda L^{T}\right)}_{i}$ represents the i-th diagonal component of matrix $L\Lambda L^{T}.$

## 3. Integrated Fault Diagnosis Framework

**Figure 2:**
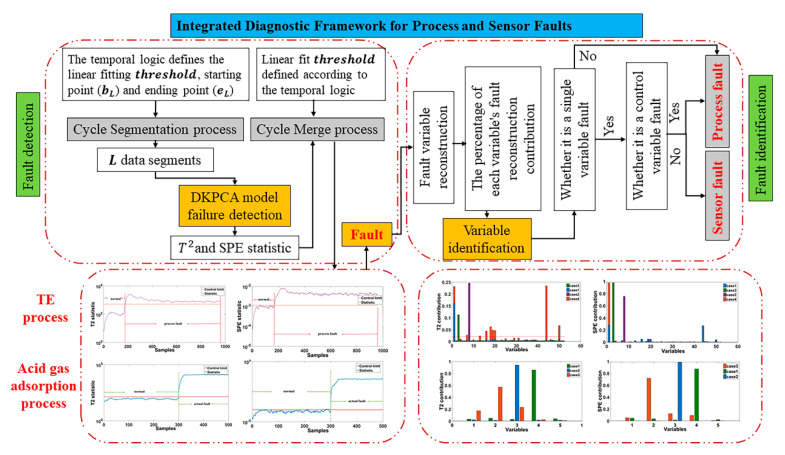
Integrated diagnosis framework for process and sensor faults.

Modern chemical process faults are mainly divided into process faults and sensor faults. Process faults are caused by large deviations in the system, and they are real faults in the system. Sensor faults are caused by sensor faults of the detection system, and the data are falsely indicated. The system remains normal. Therefore, a process fault is a multi-variable coordination, and a sensor fault is a false indication of a single measured variable. To further distinguish the types of faults when faults occur, this paper proposes an integrated fault diagnosis framework to distinguish the two types of faults in detail. The integrated fault diagnosis framework is shown in Figure 2.

The integrated fault diagnosis framework for process and sensor faults is mainly composed of two parts: fault detection and fault identification. Through the fault detection step, various faults in the system are accurately and rapidly detected. Fault identification involves identifying specific fault variables by reconstructing the variable diagram method for the detected fault and distinguishing the fault types by using the process and sensor fault characteristics.

### 3.1. Fault Detection

The fault detection part of the integrated fault diagnosis framework combines the CTA and DKPCA models. Using the cycle segmentation and cycle merge characteristics of the CTA model, the detected data are finely divided and calculated, which minimises a series of external influences such as noise, and the segmented calculation also speeds up the detection speed and accuracy. The fault information is saved to the greatest extent. The framework detects system faults timely and effectively.

**Figure 3:**
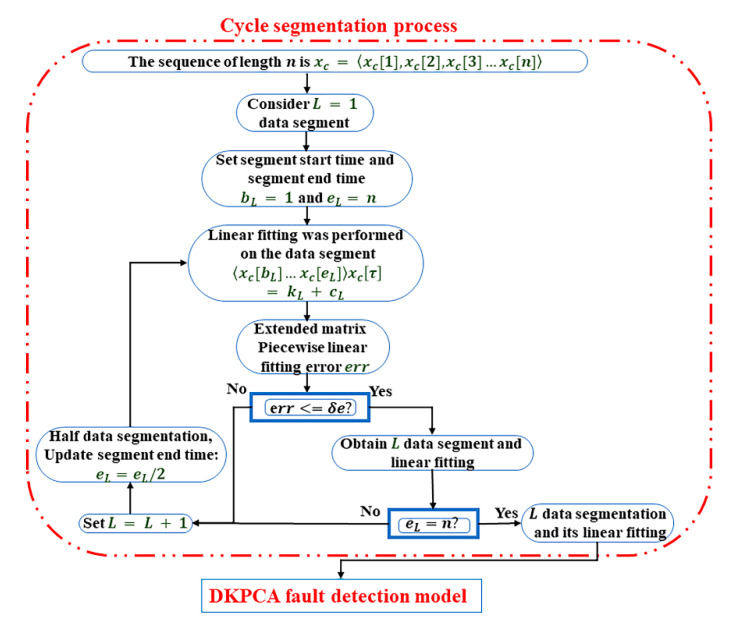
Cycle segmentation process of fault detection.

First, the detailed process of cycle segmentation is shown in Figure 3.

(1) For the process data set, first define a segment as the first segment, that is, L=1.

(2) Extract the optimal threshold $\delta_{e}$ from the temporal logic equation for the first segment of the process data set that has been determined, and define the start time $b_{1}$ and end time $e_{1}$ of the segment.

(3) Perform piecewise data fitting for segment L=1. The fitting equation is shown in Equation (10). Calculate the linear piecewise fitting error err, and determine whether the linear fitting error reaches the threshold $\delta_{e}$. If err is less than $\delta_{e}$, the segmentation stops and the current segment number is the optimal number of segments. If err is greater than $\delta_{e}$, go to step 4.

(4) Divide the data that do not meet the threshold requirement, L=L+1, define the segment end time $e_{L}=e_{L}/2$ for the new data segment, and repeat step 3 for linear fitting. Cycle segmentation calculates the linear fitting error err, until err is less than the threshold $\delta_{e}$, and enters the next judgment.

(5) Judge in the data segment that reaches the threshold $\delta_{e}$ whether the end point of the current segment reaches the end n point of the initial process data; if not, reset the start $b_{L}=e_{L}-1$ and end points $e_{L}=n$ of the unsegmented part, and enter again the step 3 of the cycle segmentation part. When the end point is reached, the cycle segmentation process ends.

**Figure 4:**
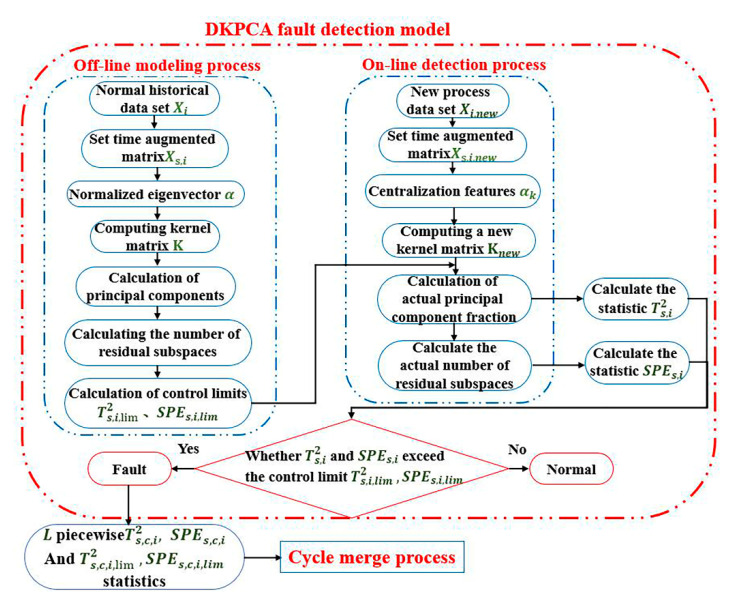
DKPCA model calculation process.

After obtaining the best segmentation data of the L segment, the segmentation data are used as the input into the DKPCA fault detection model. The fault detection process is shown in Figure 4.

The DKPCA model calculation is divided into offline modelling process and online detection process.

Offline modelling process:

(1) The offline modelling part collects the normal segmentation data set $X_{i}$, sets the superposition time t and the previous time s of each data set, and obtains the dynamic augmentation matrix $X_{s,i}$.

(2) Standardise feature vector α; the feature vector is calculated using Equation (8).

(3) Calculate the piecewise kernel matrix **K**, where the kernel function is selected as the Gaussian kernel function $K=\exp\left(-\|x-y\|{}^{2}/2\sigma^{2}\right)$.

(4) Calculate the number of principal spaces and the number of residual subspaces according to the cumulative percentage of variance (CPV) method. The equation for calculating the cumulative variance percentage is as follows:

(21) $$CPV=\frac{\sum_{i=1}^{N}\lambda_{i}}{\sum_{i=1}^{J}\lambda_{J}}$$

where λ is the characteristic value of covariance, J is the number of variables, and N is the number of principal components.

(5) Calculate the standard control limit of the no-fault condition $SPE_{i,lim},\;T_{i,lim}^{2}$, as shown in Equations (22) and (23), and enter step 5 of online detection after the calculation is completed.

(22) SPEs,i,lim=vi2miχ2mi2vi,α2

(23) $$T_{s,i,lim}^{2}=\frac{l_{i}\left(J_{i}{}^{2}-1\right)}{J_{i}\left(J_{i}-l\right)}F_{l_{i},J_{i}-l_{i},\alpha }$$

where $v_{i}$ and $m_{i}^{2}$ respectively represent the mean and variance based on $X_{s,i}$.

Online detection process:

(1) Enter the new process data set $X_{i,new}$ that needs to be detected. In the same way as step 1 of the offline stage, set the vector of the superimposition time t and the previous time s to obtain the dynamic augmentation matrix $X_{s,i,new}$.

(2) The obtained dynamic augmentation matrix is used to centralise the feature vector.

(3) Calculate the new kernel matrix $K_{new}$ by segmenting the centralised data. The kernel function used is consistent with the offline phase, which is a Gaussian kernel function.

(4) Calculate the number of principal spaces using the CPV method.

(5) Calculate nonlinear components and principal component scores. The equation is as follows: $t_{k,new}=\langle \nu_{k},\phi_{new}\left(t:t-s\right)\rangle =\sum_{i=1}^{N}\alpha_{i}^{K}\langle \phi_{i}\left(t:t-s\right),\phi \left(t:t-s\right)\rangle =\sum_{i=1}^{N}\alpha_{i}^{K}K_{new}$.

(6) Calculate the new data statistics $T_{s,i}^{2}$ and $SPE_{s,i}$ based on the reference control limits $T_{s,i,lim}^{2}$ and $SPE_{s,i,lim}$ offline non-fault conditions, as shown in Equations (24) and (25). Verify whether $T_{s,i}^{2}$ or $SPE_{s,i}$ statistics exceed the control limit $T_{s,i,lim}^{2}$,$\;SPE_{s,i,lim}$. If they exceed the control limit, it indicates that the system has a fault condition, and then enter the fault identification stage. Otherwise, it is determined to be normal, and return to step 1 to continue testing another new set of process data.

(24) $$SPE_{s,i}={\left(z^{i}\right)}^{T}\left(I_{j_{i}}-{\hat{P}}^{i}{\left({\hat{P}}^{i}\right)}^{T}\right)z^{i}\leq SPE_{s,i,lim}$$

(25) $$T_{s,i}^{2}={\left(z^{i}\right)}^{T}{\hat{P}}^{i}{\left(\Lambda^{i}\right)}^{-1}{\left({\hat{P}}^{i}\right)}^{T}z^{i}\leq T_{s,i,lim}^{2}\;$$

where $\Lambda^{i}={\left(T_{n}^{i}\right)}^{T}T_{n}^{i}/\left(N_{n}-1\right)$, and ${\hat{P}}^{i}\left(J_{i}\times l_{i}\right)$ and $T_{n}^{i}\left(N_{n}\times l_{i}\right)$ are the load matrix and score matrix of the i-th DKPCA model, respectively. $I_{J_{i}}\left(J_{i}\times J_{i}\right)$ is the unit matrix. In addition, let the column vector z(J×1) represent the current observation data point and divide z them into the corresponding L blocks based on the L variable block in the previous section, which is $z^{i}\left(J^{i}\times 1\right)\left(i=1,2,\ldots ,L\right)$.

After detecting the fault of the segmented data, the segmented data are used as the input into the cycle merge process for merging, so as to determine the confirmation of the fault of the entire process.

**Figure 5:**
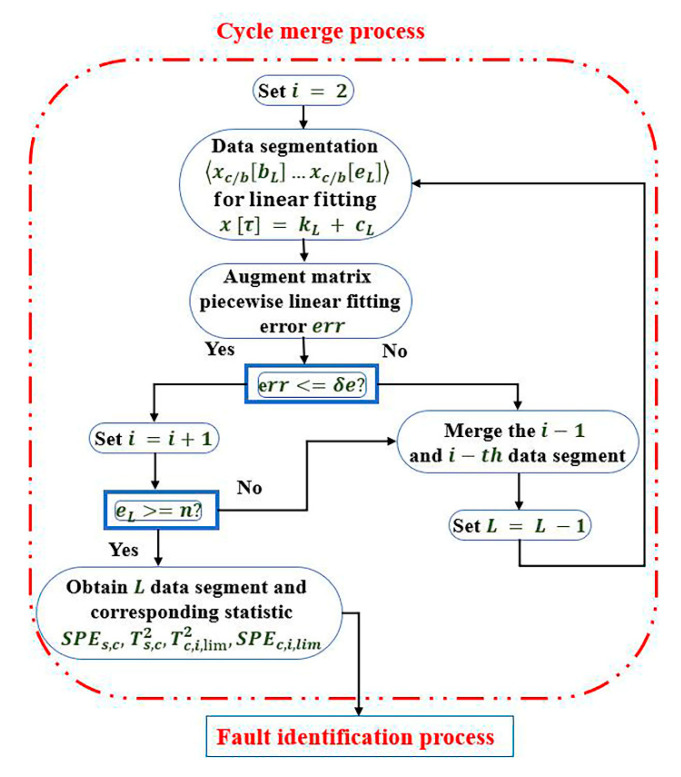
Cycle merge process of fault detection.

The detailed process of cycle merge is shown in Figure 5.

(1) The L segment $T_{i}^{2}$ and $SPE_{i}$ statistics obtained after the DKPCA model calculation part is completed are used as the input into the cycle merging stage.

(2) Set i=2, merge according to the reverse step of cycle segmentation, perform linear fitting on the first two segments, calculate the linear fitting error err, and determine whether the linear fitting error reaches the obtained threshold $\delta_{e}$.

(3) If err is greater than the threshold $\delta_{e}$, go to step 4. If the threshold $\delta_{e}$ is greater than err, merge the i−th and i−1th segments, and the total length of the data segment at this time is L−1. Return to the stage of step 2 to merge, the merged data and the next segment data are reset to the first and second segments, and the merge is repeated.

(4) When err is greater than the threshold $\delta_{e}$, it is judged whether the number of merged data segments L is less than i. If it is smaller than i, the cycle ends, and the final merged data segment is obtained. If L is greater than i, then continue to merge until L is greater than i. The cycle merge ends.

After completing the cycle merge process, the fault detection part of the integrated fault diagnosis model is completed, and the two statistics $T_{s,i}^{2}$ and $SPE_{s,i}$ detected are determined. If $T_{s,i}^{2}$ and $SPE_{s,i}$ exceed the calculated control limits $T_{s,i,lim}^{2}$ and $SPE_{s,i,lim}$, the system is considered to be faulty, and the fault identification part of the next part is further analysed.

### 3.2. Fault Identification

After the fault detection part of the integrated fault diagnosis model is completed, the fault variables in the process are identified. In this study, the RBC model is used to reconstruct and identify the fault variables, and finally, the process and sensor faults are distinguished in detail. 

**Figure 6:**
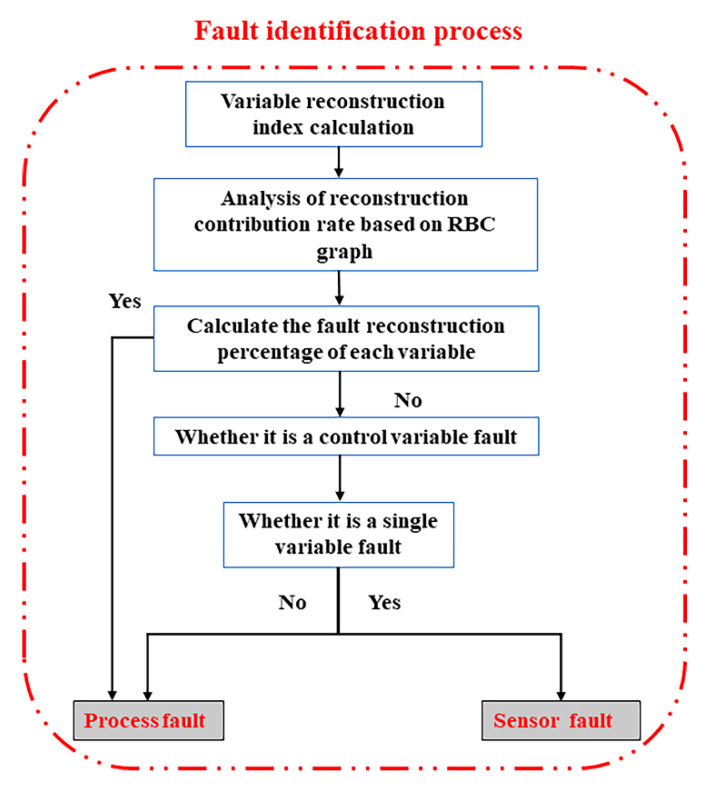
Fault identification process based on the RBC graph.

The fault identification process is shown in Figure 6.

(1) When the system detects a fault, it is used as the input to the fault identification part. The fault variable is first reconstructed using the principle of variable reconstruction, and the reconstruction index $index^{2}\left(z_{i}\right)$ is calculated.

(2) Optimise the fault parameter $f_{i}$ to capture most of the fault information.

(3) Calculate the contribution rate of variable reconstruction and calculate the percentage of the current variable contribution rate according to the multivariate statistical method. The final contribution percentage of the reconstruction variables is obtained.

According to the obtained variable reconstruction percentage, the current fault is distinguished as a process or sensor fault in detail. Process faults are generated internally by the system. They arise from a state deviation of the system control variables, which then spreads to other measured variables, and finally leads to faults. A sensor fault refers to the fault of the sensor of a measured variable, which causes false indications of the data, but the system itself does not fail. However, the operator mistakenly believes that the variable has a fault and adjusts the variable setting value, which will cause the system to fail and eventually evolve into a process fault. Therefore, it can be observed that the sensor fault is a single-variable data offset, whereas the process fault is a multi-variable coordination.

When reconstructing the contribution graph model to determine the fault variables, the possible fault variables are judged:

The RBC graph shows that a single variable exceeds the average fault contribution rate. Go to Step 2 for further judgment. If the RBC graph shows that multiple measured variables and a single control variable exceed the average fault contribution rate, it can be determined as a process fault. The corresponding control variable is the root cause of the fault.

For a situation that shows that a single variable has a fault, it is necessary to determine whether the variable is a controlled variable or a measured variable. If the fault variable is a controlled variable, it is determined that the fault is a process fault caused by a single controlled variable. If the fault variable is a measured variable, it is determined that the fault is a fault in the sensor where the variable is located, that is, a false indication of sensor data.

Based on the above detailed fault judgments, the integrated fault diagnosis model for chemical process sensors and process faults can distinguish the process or sensor faults that have occurred, even on the basis of detecting and identifying faults. It provides a technical basis for subsequent operations and helps prevent subsequent faults caused by maloperation.

## 4. Case Studies 

### 4.1. Tennessee Eastman Process

**Figure 7:**
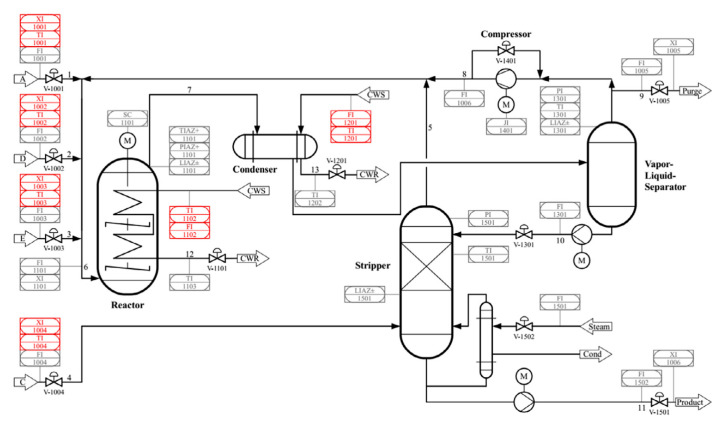
The Tennessee Eastman (TE) process.

The TE process was created by Downs and Vogel in 1993 [37] and is widely cited as a benchmark for studies in control and fault diagnosis. The flow chart of the TE process is presented in Figure 7.

The TE process includes 41 measurement variables and 11 manipulation variables. There is a strong correlation between 11 manipulation variables and their related measurement variables, while the correlation between measurement variables is relatively weak. Therefore, this process can be used as the basic process to study the correlation between variables. It includes a normal state and 21 fault states. Each state contains the training data and test data. Among them, there are 960 test data. The fault is introduced in the 160th sample. There are 21 faults in the TE process, of which 16 are known, and the remaining 5 are unknown. Fault modes 1–7 are caused by abnormal steps of some process variables, faults 8–12 are caused by random changes in some process variables; fault 13 is due to changes in reaction dynamics; and faults 14, 15, and 21 are caused by the valve being fixed in a specific position. The 21 faults are listed in Table 1.

For the TE process data, several different faults are set to distinguish the process and sensor faults. Before fault diagnosis, the basic parameters of the DKPCA model are set. The number of main components is determined by the standard that the CPV > 85% determine. The kernel width of the radial basis function was set to 800, and the confidence level was set to 95%.

Before distinguishing sensor and process faults, the advantages of the proposed model in fault detection are expressed. The number of principal components obtained by the DKPCA method is 28 for 52 variables of TE process, and the number of principal components obtained by proposed model is 26. It can be seen that the principal component obtained by proposed model includes more effective information, while there is invalid information in the principal component obtained by DKPCA method. The proposed model is more accurate in information acquisition.

In order to further quantify the accuracy and low detection delay of the proposed model, we measure its monitoring performance by fault detection rate (FDR) and time delay (TD). The Equations of FDR and TD are as follows:

(26) $$FDR=\frac{\left(control\;chart>control\;limit|F\neq 0\right)}{pre-set\;fault}\times 100\%$$

(27) $$TD=t_{d}-t_{0}$$

where $t_{d}$ is the fault detection time, and $t_{0}$ is the fault occurrent time.

The comparison results of FDR and TD statistics between CTA-DKPCA method and other methods are shown in Table 2

The results of TE process fault detection show that the proposed model improves the accuracy of fault detection and the delay of fault detection. After proving the advantages of the model for fault detection, the proposed integrated fault diagnosis framework is used to divide the possible faults in industry in detail.

This study sets four different types of fault situations to distinguish these faults as process faults or sensor faults. The selection and setting of the cases are listed in Table 3.

The first case introduces a drift fault with a drift ratio of 0.1325 at the 0 data point of variable 1: material A flow. The second case is in variable 8: in the ranges of 150–250, 400–550, and 700–850, the reactor liquid level is increased by 50%. The third case is the fault to introduce the data step at the 300th data point of the variable 3: E logistics flow. The last case is TE process simulation fault 1. Use the integrated fault diagnosis framework to diagnose the above 4 types of faults. The result of the integrated fault diagnosis is shown in Figure 5.

**Figure 8:**
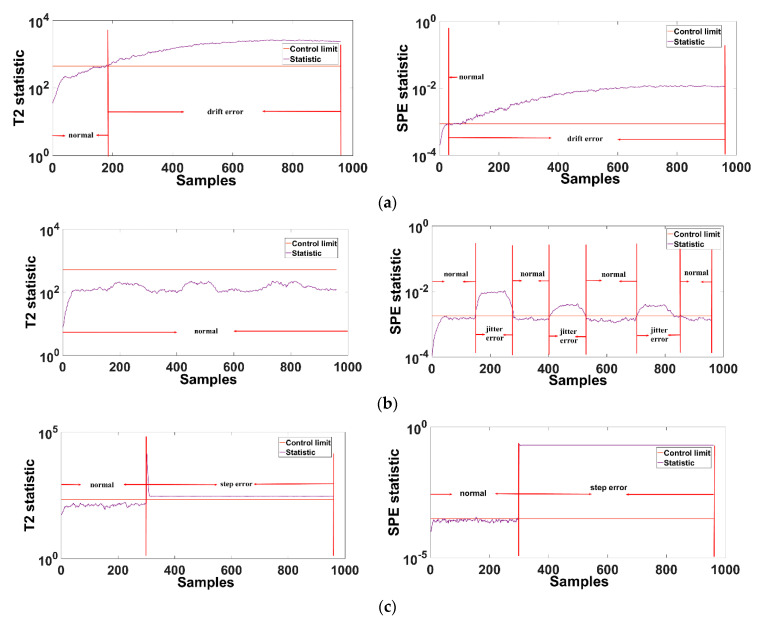

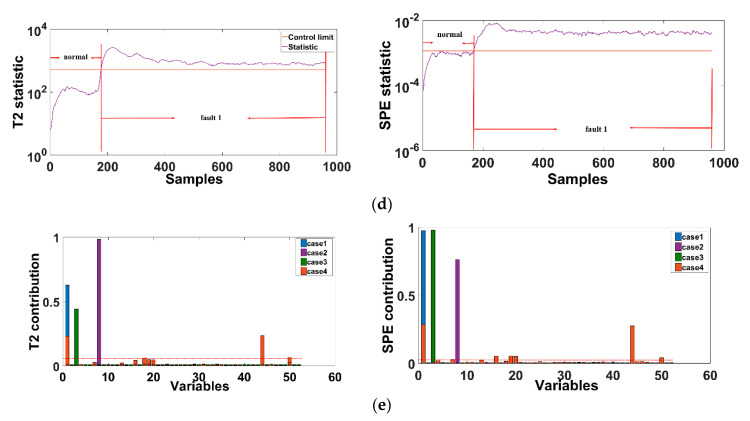
TE process fault diagnosis result. ((**a**)—case 1 fault detection result, (**b**)—case 2 fault detection result, (**c**)—case 3 fault detection result, (**d**)—case 4 fault detection result, (**e**)—fault identification result).

Figure 8a–d shows the fault detection results of the CTA-DKPCA model in the principal component subspace ($T^{2}$) and residual subspace (SPE), respectively, where (e) is the fault identification results of principal component subspace and residual subspace based on RBC. The $T^{2}$ statistic represents the macro situation of the system, that is, when there is a fault, the system can reflect the observable fault. SPE statistics represent the deviation from the normal situation in the system, that is, when the deviation occurs in the system, the normal error situation will be displayed in SPE statistics. So we can see that for the detection sensitivity of the residual subspace, the residual subspace represented by the SPE statistic is higher than the principal subspace represented by $T^{2}$ statistic. For the contribution rate of the two subspaces, the principal component space shows the macro fault condition of the system itself, and the residual subspace shows the error of the system, so the $T^{2}$ contribution rate is less than the SPE contribution rate. The error variable is observed through the SPE contribution rate, and then the $T^{2}$ contribution rate is determined as the main macro fault variable of the system.

According to the integrated fault diagnosis framework, we can obtain the synergetic characteristics of the process fault accompanying variables. The variables of sensor fault are independent of each other. The process fault caused by the fault of the control variable causes the related measurement variables to fail together. Owing to the false indication of the sensor data, the sensor fault has no transferability between the sensors, and the system remains normal.

When the drift fault ratio of case 1 is set to be small, the sensor monitoring has slow data drift, and it is determined that a fault has occurred when the tolerance limit of the sensor is reached. Therefore, it takes longer to detect the system fault with the principal component space $T^{2}$ statistic than with the residual subspace SPE statistic to detect the error in the system. From Figure 8e, it can be judged that only when variable 1 exceeds the average fault contribution of the 52 variables, and variable 1 is a measured variable, it is determined that case 1 is a sensor fault with unreliable sensor data based on the fault diagnosis result.

For case 2, the set system fluctuates. The integrated fault diagnosis result shows that the $T^{2}$ statistic does not exceed the limit, the principal component space has no fault, and the residual subspace represented by SPE detects the fault in the fluctuation interval. Therefore, it shows that there is an error in the system. The fault identification result also shows that only variable 8 exceeds the control limit, and variable 8 is a measured variable. Therefore, it can be determined that case 2 is a false indication of data fluctuation of the sensor where variable 8 is located. The system remains normal.

Case 3 is a step fault, which is an instantaneous fault, and a single variable in the system is instantly amplified. The existence of a step fault can be detected immediately, and the fault identification result shows that the variable 3 single variable exceeds the limit. Other variables are normal. Thus, it is determined that the sensor where variable 3 is located has a transient fault, which generates the false indication of the data.

Case 4 is the simulation fault 1 of the TE process. The fault diagnosis results show that both $T^{2}$ and SPE have detected the fault. The fault identification result shows that variables 1 and 44 exceed the average fault contribution rate. Variable 44 is the control variable: material A flow rate. Hence, it can be determined that case 4 is a process fault.

In addition to distinguishing the fault types of the system, the accuracy and speed of fault detection is also an important part of the fault diagnosis framework. Therefore, this study compares the FDR and TD of the four cases of the TE process with other PCA-derived algorithms. The results are presented in Table 4.

The comparison results show that the fault detection method combined with CTA and DKPCA is far superior to other PCA algorithms in terms of FDR and TD. In terms of time delay, the fault detection algorithm proposed in this paper can detect faults faster than other algorithms. The average fault diagnosis rate and time delay are 92.6% and 8.25 s.

The results show that the integrated fault diagnosis framework proposed in this paper can clearly distinguish between process faults and sensor faults. The fault detection part has excellent performance in FDR and TD. Therefore, when a fault occurs, the operator can respond in time and guide the follow-up operation according to the fault identification part.

### 4.2. Case Study of an Acid Gas Absorption Process from Natural Gas

**Figure 9:**
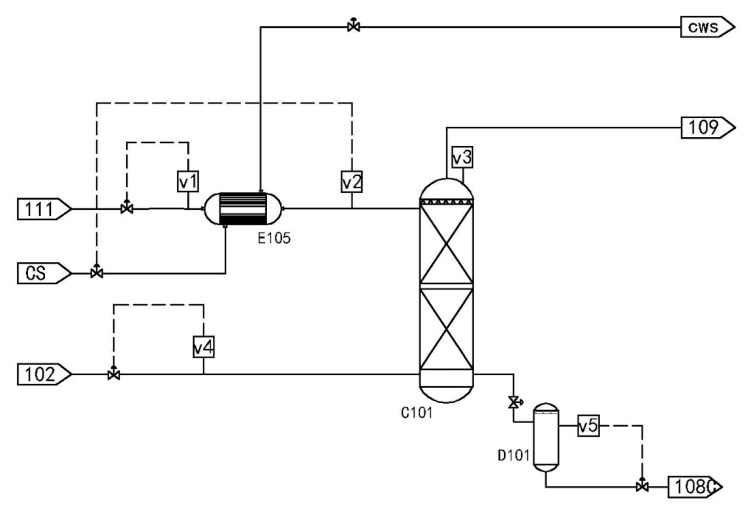
Acid gas adsorption process flow chart.

Methyldiethanolamine (MDEA) is often used as an absorbent in chemical processes to absorb acid gases. A flowchart of the typical absorption process is shown in Figure 9.

Stream 111 is the absorbent MDEA. It first exchanges heat with cooling water at room temperature through the heat exchanger E-105 and is then cooled to 21 °C, and then enters the absorption tower C-101 from the top. The raw material gas stream 102 enters the absorption tower C-101 from the bottom, flows counter currently with the absorbent MDEA, and absorbs acid gases (H_2_S and CO_2_) from the natural gas. The overhead gas of the absorption tower C-101 is natural gas containing a large amount of moisture, and then it enters the downstream dehydration system for further dehydration and purification to meet the national natural gas standards. The bottom product of the absorption tower C-101 is rich amine liquid containing acid gas. After heat exchange, the rich amine liquid enters the regeneration tower to resolve acid gas and regenerate the absorbent. According to the Piping and Instrument Diagram (P&ID) chart, the following variables: V1: absorbent MDEA volume flow rate, V2: absorption tower absorbent feed temperature, V3: absorption tower top pressure, V4: Natural gas feed flow, V5: Bottom liquid level height of absorption tower. 

Three types of faults are set in the acid gas absorption process, including drift, stuck, and actual fault. The integrated fault diagnosis framework is used to distinguish the three types of faults in detail. Examples of the fault cases are listed in Table 5.

An acid gas absorption process is provided for the three different faults. In case 1, a graded drift of the gas flow rate is monitored at the sampling point 250. In case 2, the top pressure of the absorption tower is monitored and a stuck fault is set at the sampling point 250. In case 3, an actual fault is simulated by HYSYS, that is, the temperature of the heat exchanger inlet rises to 30 °C. The integrated fault diagnosis framework is used to detect and identify the three faults, and then distinguish the fault type as a process fault or a sensor fault. The fault diagnosis results are shown in Figure 7.

**Figure 10:**
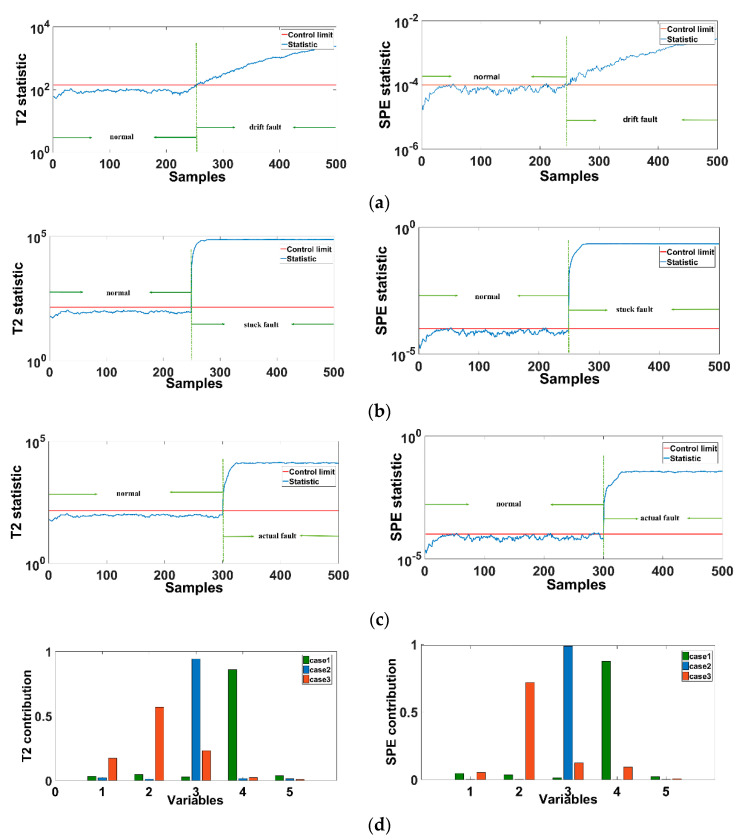
Acid gas adsorption process fault diagnosis result ((**a**)—case 1 fault detection result, (**b**)—case 2 fault detection result, (**c**)—case 3 fault detection result, (**d**)—fault identification result).

The diagnosis results for the acid gas absorption process can be obtained. For case 1, because the sensor sensitivity of the actual chemical process and the simulation process are different, the data drift of the actual process will be rapidly detected as the fault occurs, as shown in Figure 10d. The fault identification results show that variable 4 exceeds the average fault contribution rate of 20%, other variables are in a normal state, and variable 4 is the measurement variable of the condenser outlet temperature. Thus, case 1 is a sensor fault.

The actual chemical process often has a sudden stuck. The stuck is also an instantaneous fault; therefore, it will be detected instantaneously. The result of the fault variable also shows that the measured variable 3, absorption tower pressure, exceeds the average contribution rate, and other variables are normal. Hence, it is determined that case 2 is also a sensor fault.

For case 3, we introduced an actual fault of the acid gas absorption process and used the integrated fault diagnosis framework to detect and identify the fault. The detection results show that the fault can be accurately detected. The fault identification result shows that variables 1, 2, and 3 all exceed the control limit, indicating that the system has an offset condition. Therefore, it is determined that case 3 is a process fault.

In the acid gas absorption process, to determine the advantages of the proposed fault detection method in the actual chemical process, the two statistics of FDR and TD are compared with the PCA and its derivative algorithms. The comparison results are presented in Table 6.

The comparison results of various fault detection methods in the acid gas absorption case show that for actual chemical process faults, the fault detection part of the fault diagnosis framework proposed in this paper is far superior than the other algorithms in terms of fault diagnosis accuracy and speed. The FDR reached 99.73%. In cases 2 and 3 TD is 0, and the mean TD is 0.67 s, which proves that the proposed method has the best timeliness for fault detection.

Based on the above fault diagnosis results, it is shown that the CTA proposed in this paper optimises the speed and accuracy of fault detection and detects fault occurrence data points in time. The fault identification part based on the RBC graph can distinguish the chemical process sensor faults and process faults in detail. The group forms an integrated fault diagnosis framework suitable for modern chemical processes.

## 5. Conclusions

The distinction between sensor and process faults has always been an important part of chemical process fault diagnosis. This paper proposes an integrated fault diagnosis model that can effectively distinguish between sensor faults and process faults in modern chemical processes. First, a CTA is proposed to improve the calculation speed and accuracy of the model. The proposed algorithm is combined with the DKPCA for fault detection, and it is then combined with the RBC graph model to diagnose fault variables and comprehensively judge the process and sensor faults.

This study used a CTA combined with a DKPCA fault detection model to detect 18 faults in the TE process and verified its advantages in fault detection. Then, we selected four common fault types in the chemical process, including data drift, jitter, step, and actual fault. The fault detection model and the RBC fault diagnosis model were combined to distinguish the four types of faults as process faults or sensor faults in detail. Finally, based on variable correlation and fault transfer characteristics, the data drift, jitter, and step faults were determined as sensor faults, whereas the actual simulated fault was identified as process fault.

Finally, the integrated fault diagnosis model was applied to the acid gas absorption process to verify the effectiveness of the model in an actual chemical process. For the acid gas absorption process, three types of faults, namely, data drift, stuck, and actual faults, were selected to distinguish the fault types through the integrated fault diagnosis model. The results showed that data drift and sticking were sensor faults, and the actual fault was determined as the process fault. Based on the above results, this fault classification framework provides a strong foundation for the safety of chemical processes and ideas for follow-up research on chemical process fault diagnosis.

## Figures and Tables

**Table 1:** Faults for the Tennessee Eastman (TE) process.

Fault Number	Description	Type
**01**	A/C feed ratio, B composition constant (Stream4)	Step
**02**	B composition, A/C ratio constant (Stream4)	Step
**03**	D feed temperature (Stream2)	Step
**04**	Reactor cooling water inlet temperature	Step
**05**	Condenser cooling water inlet temperature	Step
**06**	A feed loss (Stream1)	Step
**07**	C header pressure loss—reduced availability (Stream4)	Step
**08**	A, B, C feed composition (Stream4)	Random
**09**	D feed temperature (Stream2)	Random
**10**	C feed temperature (Stream4)	Random
**11**	Reactor cooling water inlet temperature	Random
**12**	Condenser cooling water inlet temperature	Random
**13**	Reaction kinetics	Slow drift
**14**	Reactor cooling water valve	Sticking
**15**	Condenser cooling water valve	Sticking
**16–20**	Unknown	Unknown
**21**	Valve position constant (Stream 4)	Constant position

**Table 2:** Comparison of the FDR and TD of KPCA, 2-CLASS SVM [34], and the methods of this study in the TE process ((A) KPCA method, (B) 2-CLASS SVM method [38], (C) This study).

Fault No	KPCA		2-CLASS SVM		This Study	
	FDR (%)	TD (s)	FDR (%)	TD (s)	FDR (%)	TD (s)
**1**	83.0	20.0	**99.9**	6.0	99.2	**3.0**
**2**	90.0	78.0	97.8	57.0	**98.5**	**5.0**
**4**	75.0	26.0	100.0	3.0	**100.0**	**3.0**
**5**	80.0	18.0	99.9	6.0	**100.0**	**4.0**
**6**	77.0	10.0	**100.0**	3.0	98.4	**3.0**
**7**	75.0	14.0	**100.0**	3.0	99.1	**3.0**
**8**	72.0	112.0	95.8	60.0	**100.0**	**10.0**
**10**	68.0	40.0	85.8	12.0	**100.0**	**6.0**
**11**	81.0	25.0	**96.6**	3.0	92.0	**3.0**
**12**	59.0	36.0	**100.0**	3.0	99.0	**2.0**
**13**	75.0	259.0	91.9	153.0	**92.0**	**30.0**
**14**	55.0	21.0	**100.0**	3.0	99.5	**3.0**
**16**	66.0	29.0	**96.9**	**3.0**	91.2	4.0
**17**	71.0	99.0	92.9	72.0	**98.9**	**3.0**
**18**	81.0	378.0	90.0	231.0	**93.5**	**4.0**
**19**	52.0	45.0	88.5	3.0	**92.5**	**3.0**
**20**	70.0	77.0	85.0	45.0	**91.5**	**5.0**
**21**	51.0	12.0	100.0	3.0	**100.0**	**3.0**
**Mean**	71.2	71.61	95.6	37.17	**96.96**	**5.39**

**Table 3:** Four faults in TE process.

Case Number	Fault Situation	Description
**1**	Single variable data drift	Variable 1, data points 0–960: set the drift ratio of 0.1325 to the data
**2**	Large-scale jitter of process data	Variable 8, data points 150–250, 400–550, and 700–850: set jitter up by 50%
**3**	Single variable data step	Variable 3, set a step fault at data point 300
**4**	Actual fault	TE process fault 1

**Table 4:** Comparison with PCA, KPCA, DKPCA in FDR and TD.

Case No	PCA		KPCA		DKPCA		This Study	
	FDR (%)	TD (s)	FDR (%)	TD (s)	FDR (%)	TD (s)	FDR (%)	TD (s)
**1**	48.6	278.0	67.6	55.0	72.5	50.0	**79.9**	**25.0**
**2**	16.5	125.0	78.5	65.0	89.2	25.0	**91.3**	**5.0**
**3**	83.0	12.0	95.0	10.0	98.0	2.0	**100**	**0.0**
**4**	31.0	35.0	83.0	20.0	91.0	11.0	**99.2**	**3.0**
**Mean**	44.78	112.5	81.03	37.5	87.68	22.0	**92.6**	**8.25**

**Table 5:** Three faults in acid gas adsorption process.

Case Number	Fault Situation	Description
**1**	data drift	Set a slowly varying drift of the natural gas feed flow (V4) at data point 250
**2**	data stuck	Set a stuck of the absorption tower top pressure (V3) at data point 250
**3**	Fault 1	Heat exchanger inlet temperature rises to 31 ℃ at data point 300

**Table 6:** Comparison with PCA, KPCA, DKPCA in FDR and TD.

Case No	PCA		KPCA		DKPCA		This Study	
	FDR (%)	TD (s)	FDR (%)	TD (s)	FDR (%)	TD (s)	FDR (%)	TD (s)
**1**	56.1	24.0	77.0	15.0	90.1	5.0	**99.2**	**2.0**
**2**	78.9	12.0	89.0	8.0	98.2	4.0	**100.0**	**0.0**
**3**	65.5	34.0	80.0	19.0	89.5	6.0	**100.0**	**0.0**
**Mean**	66.83	23.33	82.0	14.0	92.6	5.0	**99.73**	**0.67**

## Data Availability

Data available on request due to restrictions eg privacy or ethical.
